# Supplementary material for: Engrafted parenchymal brain macrophages differ from microglia in transcriptome, chromatin landscape and response to challenge
Source: Nat Commun. 2018 Dec 6;9:5206. doi: 10.1038/s41467-018-07548-5 (PMC6284018; doi:10.1038/s41467-018-07548-5)
Supplement: Supplementary file 1 — Supplementary Information [file 41467_2018_7548_MOESM1_ESM.pdf]

Supplementary Information

Engrafted brain macrophages differ from host microglia in transcriptome, chromatin landscape and response to challenge

Shemer et al.

Supplementary Figure 1

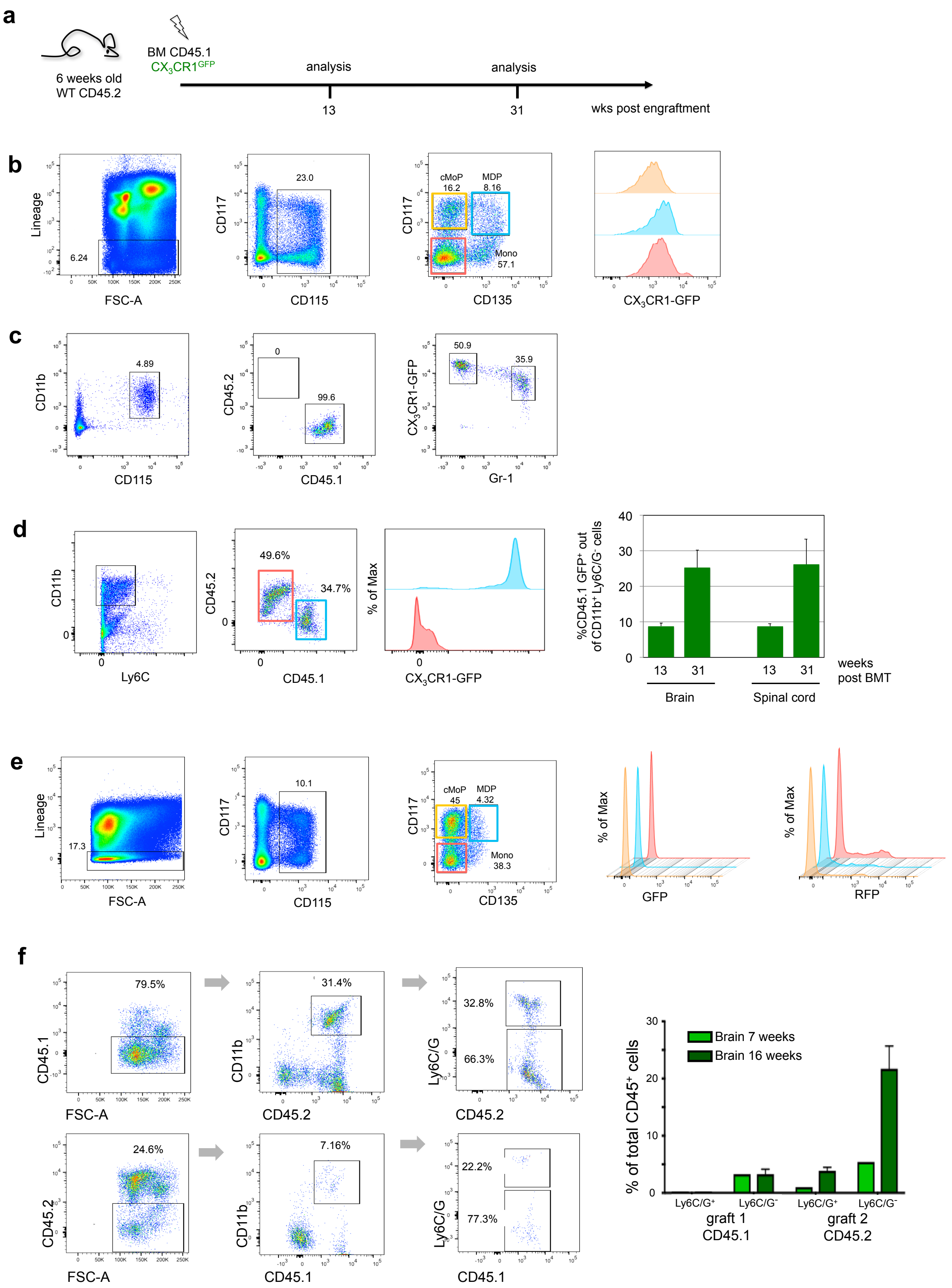

Supplementary Figure 1

BM-derived parenchymal brain macrophages accumulate over time post-irradiation.

(a) Schematic of BM transfer protocol.

(b) Flow cytometry analysis of myeloid BM progenitor compartment of chimeras 13 weeks post-BMT.

(c) Flow cytometry analysis of blood monocytes of chimeras 13 weeks post-BMT.

(d) Flow cytometry analysis of brain cells of chimeras 13 weeks post-BMT. Of note, $Ly6C^-$ $CD11b^+$ cells comprise host microglia (CD45.2) and BM-derived cells (GFP^+ CD45.1).

(e) Analysis of myeloid BM progenitor compartment of chimeras 7 weeks post second BMT. Note that none of the myeloid progenitors expressed GFP, while MDP, cMOP and monocytes do show RFP signal.

(f) Flow cytometric analysis of brain cells 16 weeks post second BMT for distribution of first and second graft-derived myeloid cells ($CD11b^+$ $Ly6C/G^+$ and $CD11b^+$ $Ly6C/G^-$). Bar graph shows distribution of cells derived from first and second graft out of the total $CD45^+$ cells in brains at two time points. Data are a summary of 3 mice per time point.

Supplementary Figure 2

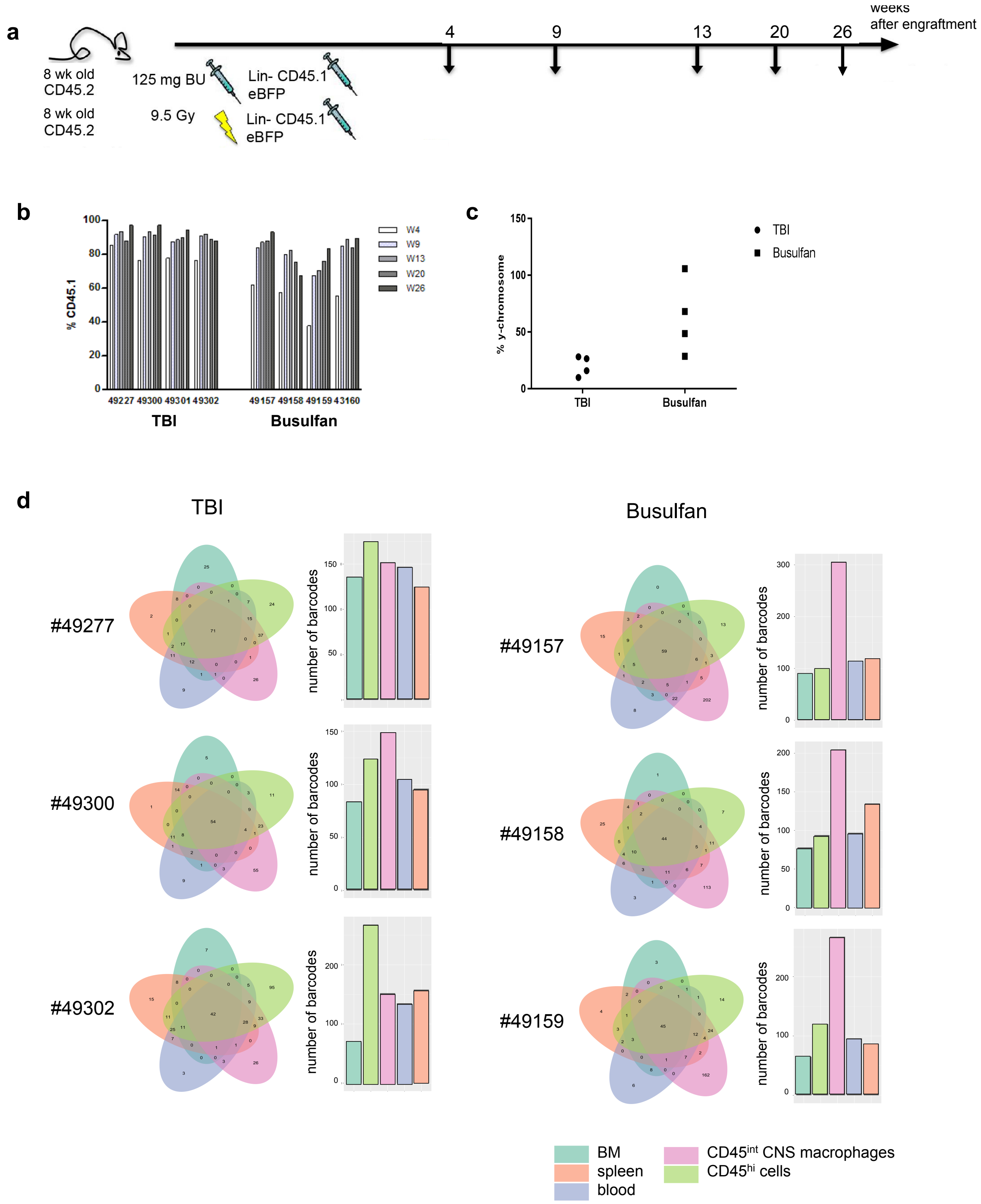

Supplementary Figure 2

Clonal analyses on TBI- and Busulfan-treated animals.

(a) Experimental set-up: Lineage-negative cells from 8-weeks old male donors were transduced with lentiviral vectors carrying eBFP as a fluorescent reporter, as well as a BC32-eBFP barcode system [1], prior to transplantation into 8-weeks old female recipients conditioned either with total body irradiation (TBI) or chemotherapy (Busulfan). Peripheral blood samples were taken every 4-6 weeks post transplantation to monitor engraftment. 6 months post transplantation, animals were sacrificed and peripheral blood, BM, spleen and the brain were taken for clonal analyses.

(b) Flow cytometry on peripheral blood samples to measure the chimerism.

(c) Chimerism of sorted parenchymal brain macrophages as detected by Y-chromosome-specific digital droplet PCR.

(d) Barcode analyses on DNA extracted from peripheral blood, BM, spleen and sorted $CD45^{int}$ brain macrophages and $CD45^{high}$ CNS cells. Numbers of barcodes for the samples are shown in the bar plots and shared barcodes of the respective sample are displayed in the Venn diagrams.

Supplementary Figure 3

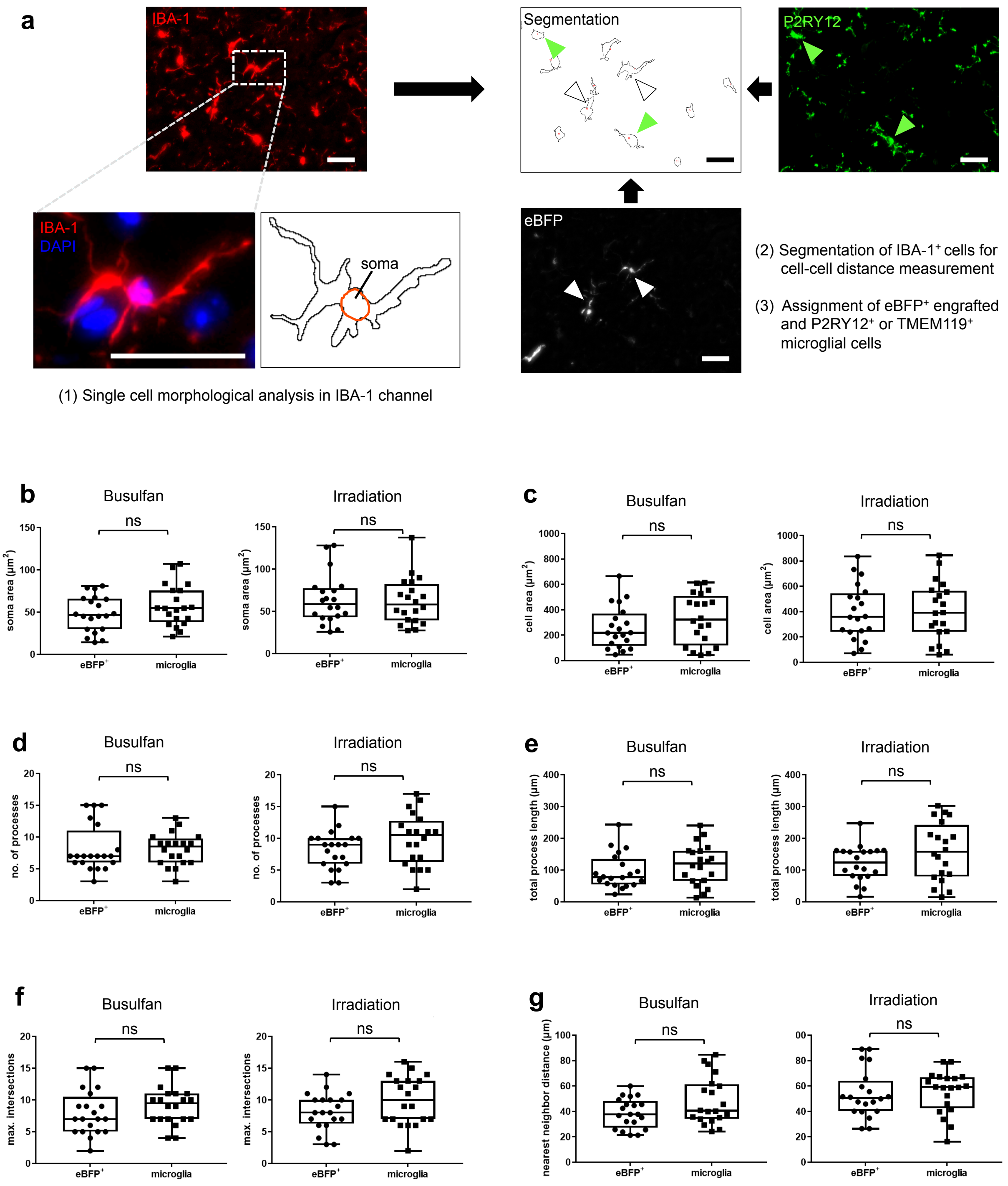

Supplementary Figure 3

Measurements of host and donor cells from recipients of lineage-negative BM that carrying lentiviral constructs conferring eBFP expression.

a) Workflow of measurements showing that all cortical microglial cells were segmented and randomly sampled for single cell morphological analysis in the Iba-1 (red) channel. Nuclear DAPI counterstain (blue) was used to indicate soma location. For unblinding of cell type, immunolabel for P2RY12 (green; filled arrowheads) or TMEM119 (not shown) identified host microglial cells, while eBFP expression (white; open arrowheads) indicated donor cells. Scale bars, 30 µm.

b-g) In maximum intensity projection images, single cells were analyzed for (b) area of cell soma, (c) area covered by soma and processes, (d) number of processes, (e) total length of the processes, (f) maximum number of intersections of the processes determined by Sholl analysis, and (g) nearest neighbor intercellular distance. All data sets are normally distributed (D'Agostino-Pearson test, $p > 0.05$), except the total process length of $eBFP^+$ donor cells in Busulfan-conditioned mice (D'Agostino-Pearson test, $p = 0.0358$). For each brain conditioning paradigm (Busulfan, TBI), the parameters of 20 cells per cell type were compared by two-tailed unpaired t-test. ns, not significant.

Supplementary Figure 4

a untreated animals

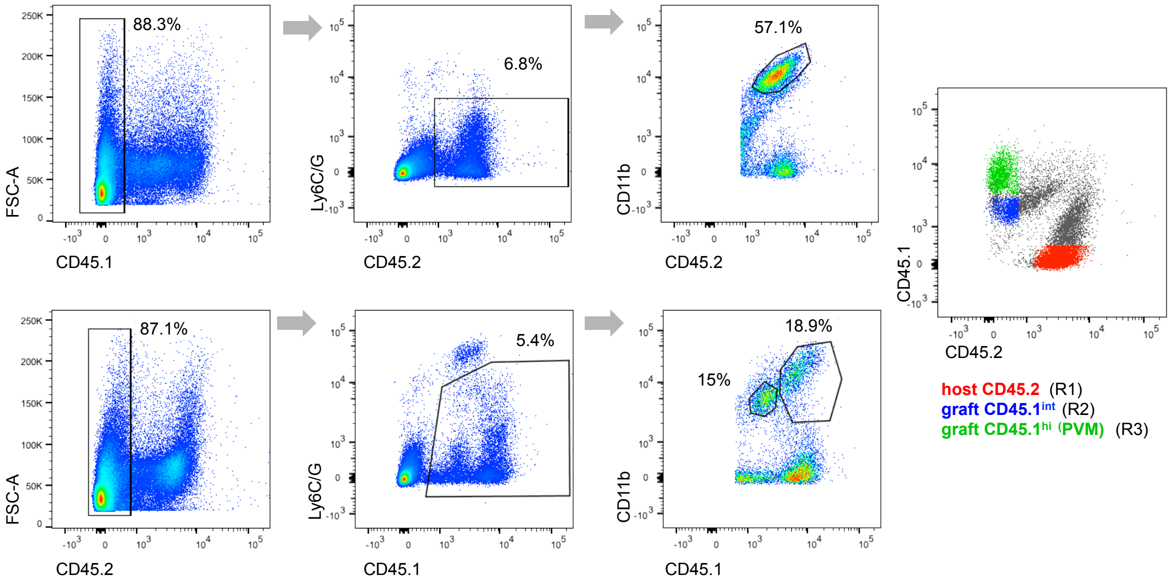

b 12hrs after i.p. LPS treatment

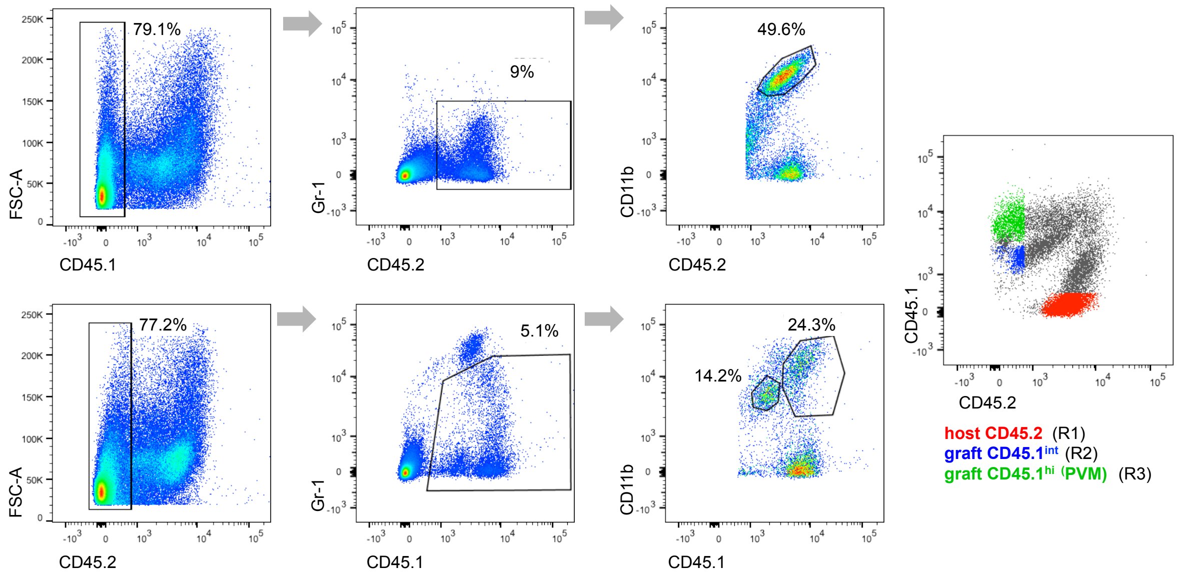

Flow cytometry analysis of chimeras used for isolation of cells for transcriptome epigenome analysis

a) Representative flow cytometry analysis of brain of [CD45.1 > CD45.2] chimera, 9 months after engraftment. Note presence of graft derived- parenchymal macrophages ($CD45.1^{int}$) and perivascular macrophages ($CD45.1^{hi}$), but absence of the latter from the $CD45.1^{+}$ host compartment. Dot blot on the right indicates respective population in the sort gate (Fig. 3a)

b) Representative flow cytometry analysis of brain of [CD45.1 > CD45.2] chimera, 9 months after engraftment and 12 hours after intra-peritoneal LPS treatment. Dot blot on the right indicates respective population in the sort gate (Fig. 3a).

Supplementary Figure 5

a

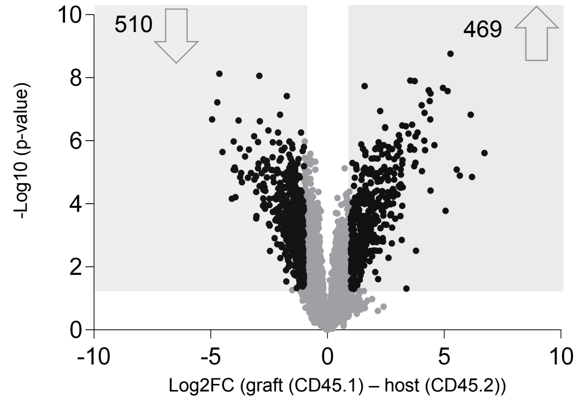

b

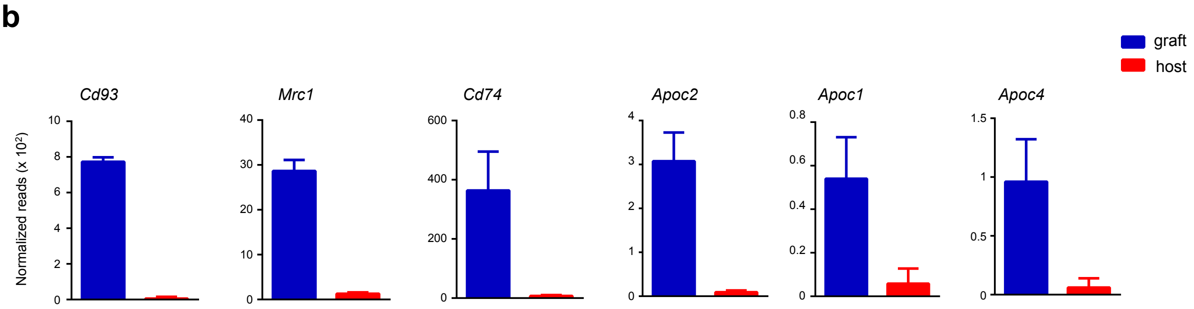

c

	host 1	host 2	host 3	host 4	host 5	graft 1	graft 2	graft 3	graft 4	graft 5	non-irradiated MG 1	non-irradiated MG 2	non-irradiated MG 3
host 1	1												
host 2	0.988472	1											
host 3	0.974321	0.993990647	1										
host 4	0.986449	0.997411836	0.994455	1									
host 5	0.959172	0.986139	0.989819	0.988375	1								
graft 1	0.431397	0.446284	0.450021	0.433159	0.420734	1							
graft 2	0.20391	0.220991	0.233341	0.211148	0.204299	0.9501446	1						
graft 3	0.533481	0.542920	0.539857	0.530383	0.512875	0.961605405	0.857912195	1					
graft 4	0.374389	0.389396	0.395167	0.377384	0.365958	0.95722337	0.902023319	0.922959506	1				
graft 5	0.331718	0.346608	0.352759	0.336069929	0.326516414	0.97286904	0.939637803	0.928676741	0.986788168	1			
non-irradiated MG 1	0.958512597	0.979276061	0.976153859	0.977068043	0.976856912	0.52408286	0.311645525	0.603922276	0.459988027	0.429121818	1		
non-irradiated MG 2	0.95303496	0.975811799	0.971227357	0.973408692	0.978910242	0.512409546	0.299483711	0.596356184	0.44822863	0.41930029	0.99712232	1	
non-irradiated MG 3	0.960912618	0.98000665	0.977206527	0.97853232	0.975458965	0.530242354	0.318278007	0.611133817	0.465887866	0.433369758	0.99776659	0.995292889	1

d

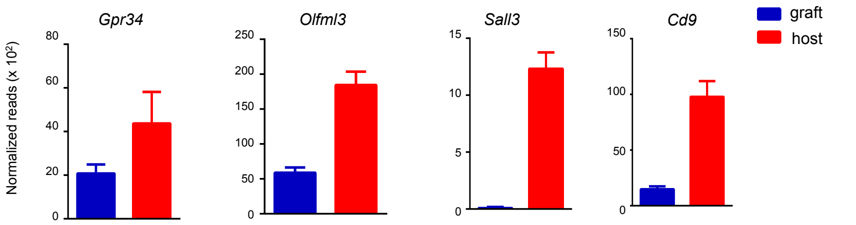

Supplementary Figure 5

Comparison of transcriptomes of engrafted cells and host microglia.

a) Volcano plot of RNA seq data of samples acquired in Fig. 3a, showing up- and down regulated genes in the graft-derived cells relative to host microglia. Analysis was restricted to expressed genes. Dark dots indicate genes showing a 2-fold difference and yielding p-values <0.05 between the two populations, N=4.

b) Examples of gene expression enriched in engrafted cells. Graphs show normalized reads from RNA seq data of samples acquired in Fig. 3a, N=4.

c) Correlation matrix of transcriptomes of engrafted cells and host microglia with transcriptome of microglia isolated from brains of age-matched non-irradiated C57BL/6 WT mice.

d) Examples of gene expression enriched in host microglia. Graphs show normalized reads from RNA seq data of samples acquired in Fig. 3a, N=4.

Supplementary Figure 6

a

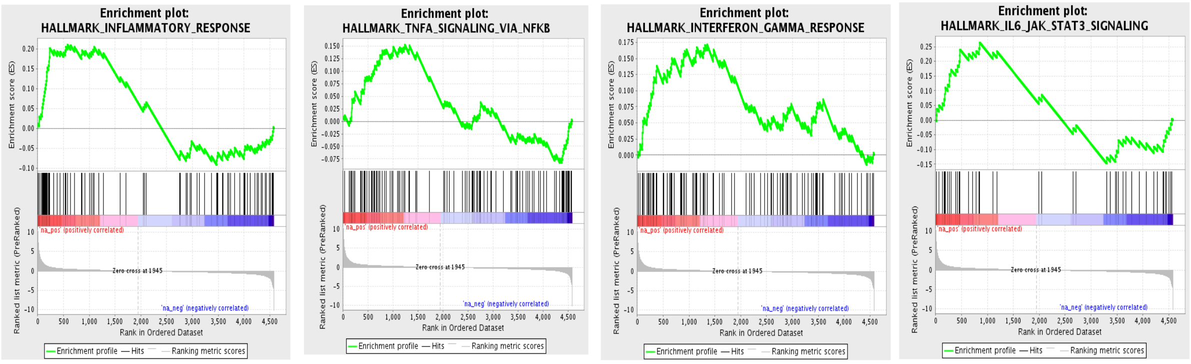

FDR q –value 0.005230476

FDR q –value 0.0031200416

FDR q –value 0.00117948055

FDR q –value 0.009422488

b

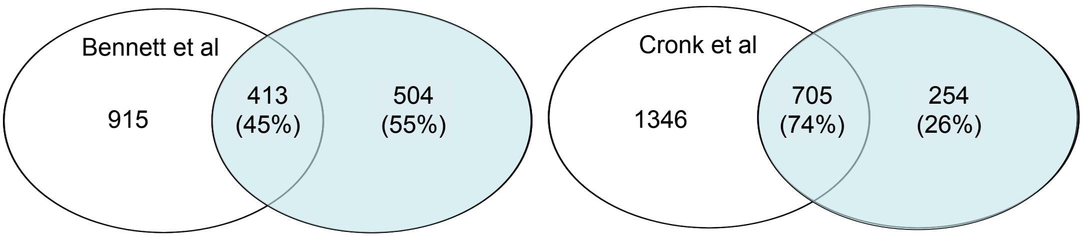

Pathway analysis and transcriptome comparisons

a) GSEA analysis of transcriptomes of HSC-derived engrafted brain macrophages and microglia.

b) Comparative analysis of lists of genes differentially expressed by graft-derived brain macrophages and host microglia in this study to the data retrieved from the study by Kipnis and colleagues [2] and Bennett et al. [3]. Blue area represents DEG of engrafted macrophages and host microglia of this study.

Supplementary Figure 7

a

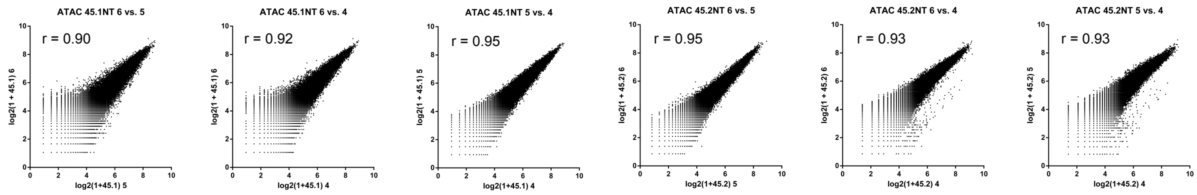

b

	-log10 (p-value)	
**motif**	graft	host
BCL11A	40.04648	23.06527
CEBPA	32.43423	21.85961
CTCF	50	50
CTCFL	50	41.91764
ETV6	50	50
Ets-related_factors_1_merged	43.98128	33.19889
Ets-related_factors_3_merged	50	50
Regulators_of_differentiation_1_merged	50	50
Runt-related_factors_2_merged	38.75022	24.48596
ZEB1	50	49.99995

ATACseq data of engrafted cells and host microglia isolated from brains of unchallenged BM chimeras.

a) Correlation analysis of ATACseq samples prepared from sorted grafted cells and host microglia.

b) List of highly abundant motifs identified in sequences coinciding with ATACseq peaks (-log10 (p-value) > 66.37). See Supplementary Source Data.

Supplementary Figure 8

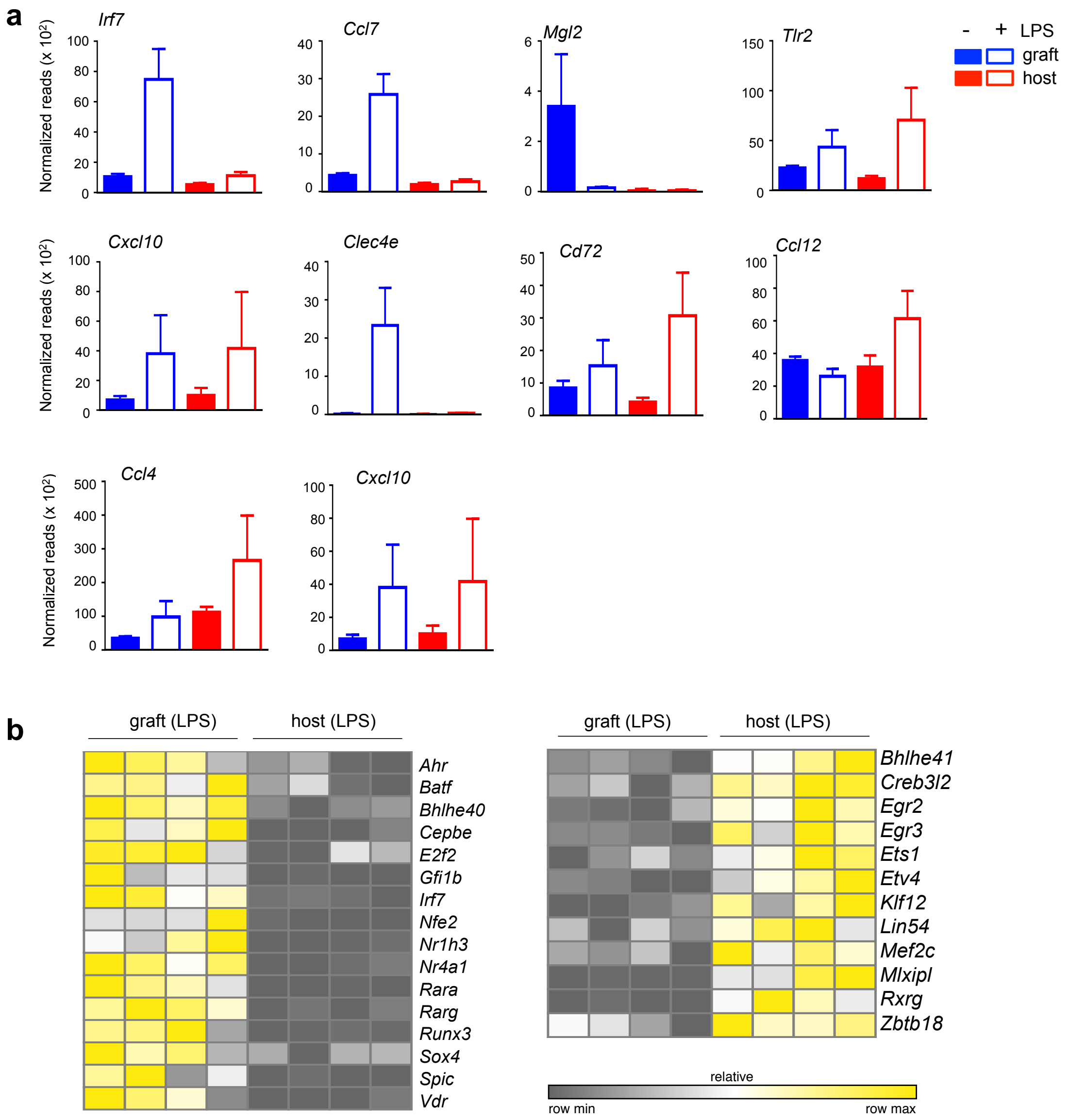

Comparison of engrafted cells and host microglia isolated from brains of BM chimeras subjected to peripheral LPS challenge.

a) Examples of genes expressed by grafted cells and host microglia in steady state and 12h post-LPS. See Supplementary Source Data.

b) Heatmap showing differential TF expression profiles of grafted cells and host microglia isolated from LPS challenged chimeras.

Supplementary Figure 9

a

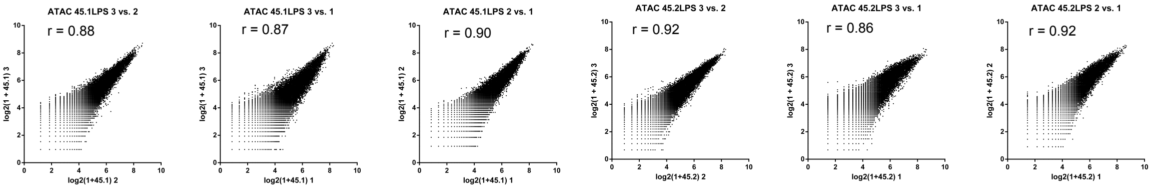

b

-log10 (p-value)

**motif**	**graft**	**host**
CEBPA	49.329186	49.9673163
CTCF	50	50
ETV6	23.1960544	35.2170259
Ets-related_factors_3_merged	50	50
ZEB1	41.0636486	39.8957154

ATACseq data of engrafted cells and microglia isolated from mice challenged with LPS

a) Correlation analysis of ATACseq samples prepared from sorted grafted cells and host microglia isolated from LPS challenged BM chimeras.

b) List of highly abundant motifs identified in sequences coinciding with ATACseq peaks (-log10 (p-value) > 50.86) in grafted cells and microglia isolated from LPS challenged BM chimeras. See Supplementary Source Data.

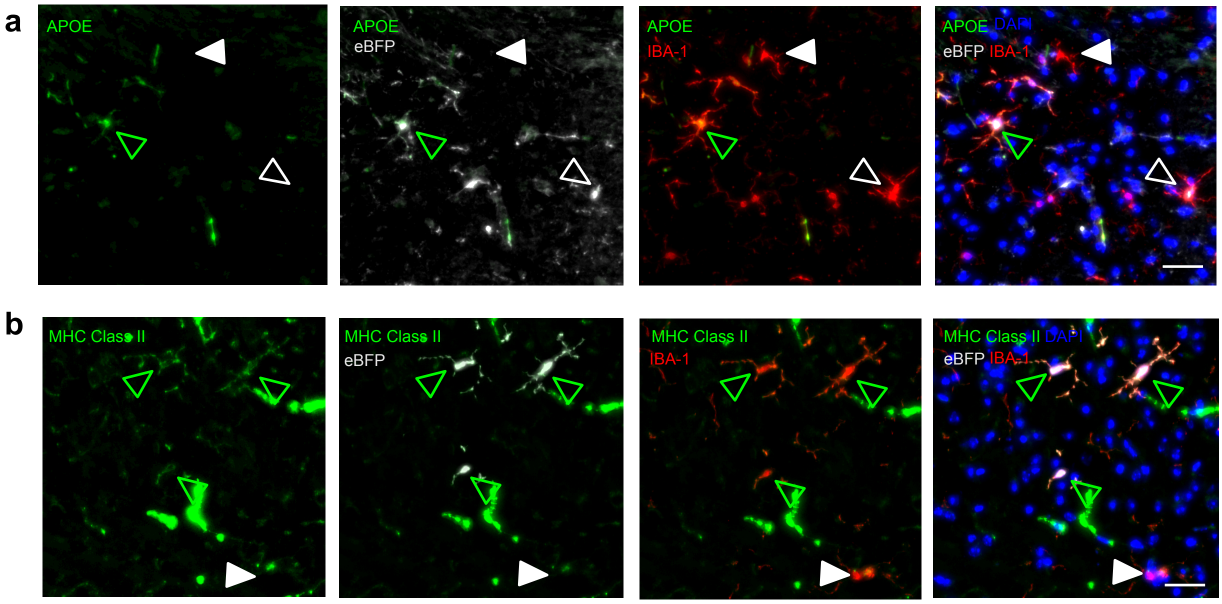

Supplementary Figure 10

Comparative protein expression analysis of grafted cells and host microglia in mouse chimeras.
Expression of the graft-specific markers APOE (a) and MHC class II (b) (green) in the cortex of mice that received lineage negative BM carrying the lentiviral construct conferring eBFP expression. Host (filled) and donor (open) cells are indicated with respective arrowheads. Iba-1 immuno-histochemistry for microglia (red). DAPI nuclear counterstain (blue). Scale bars, 30 µm.

References

1. Aranyossy, T., Thielecke, L., Glauche, I., Fehse, B. & Cornils, K. Genetic Barcodes Facilitate Competitive Clonal Analyses In Vivo. *Human Gene Therapy* **28,** 926–937 (2017).
2. Cronk, J. C. *et al.* Peripherally derived macrophages can engraft the brain independent of irradiation and maintain an identity distinct from microglia. *J. Exp. Med.* **47,** jem.20180247–21 (2018).
3. Bennett, F. C. *et al.* A Combination of Ontogeny and CNS Environment Establishes Microglial Identity. *Neuron* 1–23 (2018). doi:10.1016/j.neuron.2018.05.014

Supplementary Table 1

a

Upstream Regulator	Expr Log Ratio (graft / host)	Predicted Activation State	Activation z-score	p-value of overlap	Mechanistic Network
TGFB1	-0.464		-0.029	1.58E-28	276 (18)
LPS			1.936	2.40E-28	299 (17)
IL4			0.083	4.74E-24	254 (15)
IFNG			1.713	1.08E-22	241 (19)
IL10RA	-0.334		-1.72	7.44E-22	193 (19)
TNF	-1.59		0.107	5.19E-21	264 (16)
dexamethasone			0.274	4.86E-20	285 (19)
IL6	-2.33		1.384	2.36E-19	261 (17)
APP	0.337		0.192	2.51E-19	244 (17)
CSF3	0		0.791	3.61E-17	234 (22)
CSF2			1.015	4.18E-17	283 (19)
IL10	5.02		-1.358	6.89E-17	237 (19)
poly rI:rC-RNA		Activated	2.711	9.02E-17	235 (18)
IL1B	1.37		1.803	1.15E-16	247 (16)
IL2		Activated	2.071	3.18E-16	251 (18)

b

Upstream Regulator	Expr Log Ratio (graft / host)	Predicted Activation State	Activation z-score	p-value of overlap	Mechanistic Network
LPS		Activated	6.292	2.38E-64	324 (15)
IFNG		Activated	5.652	4.75E-53	289 (14)
TNF	-1.71	Activated	5.042	2.62E-43	277 (14)
IL1B	1.03	Activated	4.395	1.70E-41	322 (14)
TGFB1	0.332		0.544	3.00E-37	311 (19)
IL4		Activated	2.937	6.06E-33	309 (15)
dexamethasone			1.166	1.06E-32	321 (16)
IL10RA	-0.159	Inhibited	-4.971	8.40E-31	217 (13)
TNFSF11		Activated	2.416	1.43E-29	259 (14)
IL13			1.037	1.87E-29	267 (15)
STAT1	0.536	Activated	4.556	5.92E-29	262 (16)
STAT3	0.14		1.69	9.19E-29	275 (18)
IL6	-0.955	Activated	3.462	2.84E-28	296 (16)
IRF3	-0.219	Activated	4.755	1.28E-27	206 (13)
E. coli B4 LPS		Activated	3.406	4.25E-27	243 (17)

c

Upstream Regulator	Expr Log Ratio (graft NT / LPS)	Predicted Activation State	Activation z-score	p-value of overlap	Mechanistic Network
LPS		Activated	10.409	2.79E-76	515 (13)
IFNG		Activated	7.137	5.98E-63	447 (13)
IL1B	2.85	Activated	7.232	1.47E-55	455 (13)
TNF	1.54	Activated	8.266	3.94E-51	461 (13)
IL4		Activated	3.135	5.43E-43	527 (17)
TGFB1	-0.71	Activated	3.15	1.20E-42	510 (18)
dexamethasone			0.194	1.84E-38	602 (17)
TNFSF11		Activated	4.885	1.67E-34	462 (13)
IL10RA	-0.585	Inhibited	-6.758	1.64E-30	344 (16)
poly rI:rC-RNA		Activated	6.779	2.74E-29	461 (16)
beta-estradiol		Activated	2.073	2.10E-28	649 (20)
TP53	-0.9		1.761	3.08E-28	489 (19)
CD40LG			1.886	7.21E-28	409 (15)
IL6	2.54	Activated	3.755	1.02E-27	458 (13)
PD98059		Inhibited	-3.87	2.02E-27	550 (19)

d

Upstream Regulator	Expr Log Ratio (host NT/ LPS)	Predicted Activation State	Activation z-score	p-value of overlap	Mechanistic Network
LPS		Activated	7.457	5.68E-33	367 (14)
IL1B	3.19	Activated	5.235	1.20E-26	315 (13)
IFNG		Activated	5.276	2.05E-25	317 (14)
TNFSF11		Activated	3.984	2.88E-23	290 (11)
poly rI:rC-RNA		Activated	5.368	1.04E-22	323 (15)
TNF	1.66	Activated	5.972	1.65E-21	310 (13)
TLR4	-1.21	Activated	4.144	1.29E-19	283 (14)
TGFB1	-1.51	Activated	2.604	3.02E-19	374 (16)
dexamethasone			0.666	5.94E-19	385 (17)
E. coli B5 LPS		Activated	4.473	1.72E-18	308 (13)
IL4			-0.634	6.98E-18	325 (17)
S. minnesota R595 LPSs		Activated	4.95	7.57E-18	285 (14)
TICAM1	-0.095	Activated	5.04	1.02E-16	227 (16)
methylprednisolone		Activated	2.942	1.54E-16	388 (19)
APP	1.26	Activated	4.786	2.25E-16	371 (16)

Ingenuity Pathway analyses (IPA)

a) IPA analysis of genes differentially expressed by engrafted macrophages and host microglia isolated from untreated BM chimeras.

b) IPA of transcriptomes of HSC-derived engrafted parenchymal brain macrophages and host microglia isolated from animals 12hrs after peripheral LPS challenge.

c) IPA of genes differentially expressed by engrafted macrophages isolated from untreated BM chimeras or animals 12hrs after peripheral LPS challenge.

d) IPA of genes differentially expressed by host microglia isolated from untreated BM chimeras or animals 12hrs after peripheral LPS challenge.
